# Movement Ecology of Adult Male Threatened Katipō ( *Latrodectus katipo* ) and Invasive False Katipō ( *Steatoda capensis* )

**DOI:** 10.1002/ece3.72232

**Published:** 2025-10-08

**Authors:** James J. Roberts, Anne Wignall, Dianne H. Brunton

**Affiliations:** ^1^ School of Biological Sciences University of Auckland Auckland New Zealand; ^2^ Ecology New Zealand Limited Auckland New Zealand; ^3^ School of Natural Sciences, College of Science Massey University Auckland New Zealand

**Keywords:** behavioural ecology, conservation, exploration, habitat use, invasive species

## Abstract

Studies in movement ecology are crucial for understanding how physical performance affects an animal's ability to find resources, including new ranges or mating opportunities. Invasive species frequently exhibit high levels of boldness and exploratory behaviour, enhancing their ability to establish and spread in new environments. Our study compared the locomotor performance and exploration behaviour of adult male endemic katipō ( *Latrodectus katipo* ) and adult male invasive false katipō ( *Steatoda capensis* ) spiders collected from a sympatric population. We compared the locomotory and exploration behaviour of these two spider species within the context of differences in their distribution patterns and conservation classification. We conducted three laboratory assays (maze, pole and track) to compare exploration, climbing and running behaviours, respectively. We found that male false katipō are more exploratory than katipō. This aligns with the false katipō's broader habitat range. Male katipō and false katipō had similar pole climbing behaviours, which may reflect the similar use of vertical space within refuges of the two species. False katipō completed the running track faster and more often without stopping when compared to katipō. In contrast, katipō would occasionally freeze while being followed down the track with a paintbrush. Our study provides insight into the comparative locomotor performance of katipō and false katipō, highlighting the potential impacts of physical and behavioural traits on invasive species success and native species decline.

## Graphical abstract

Locomotor performance influences an animal's ability to find resources, including new habitats or mates. We compared the movement and exploration behaviours of adult male endemic katipō ( *Latrodectus katipo* ) and invasive false katipō ( *Steatoda capensis* ). False katipō exhibited greater exploratory behaviour and faster running speeds, while climbing behaviours were similar between species. These findings suggest that behavioural traits may contribute to the invasive success of false katipō and potential impacts on katipō populations.

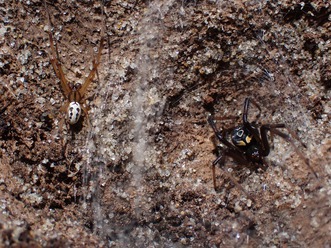

## 1 Introduction

Invasive species often have different locomotor behaviours that provide them an advantage over native species. These include increased boldness and greater exploratory tendencies (Baxter‐Gilbert et al. 2021; Chuang and Riechert 2021; Liebl and Martin 2012; Nordberg et al. 2021). Boldness and exploration are important components of an organism's movement ecology (Réale et al. 2007; Reader 2015). These traits develop in response to selective pressures in the species' native habitats, where bold or exploratory individuals may have greater survival and reproductive success (Chapple et al. 2022; Chuang and Riechert 2021; Mowery et al. 2021). Such behaviours can support establishment in novel environments by helping individuals locate resources, evade predators and rapidly colonise habitats (Chapple et al. 2012; Damas‐Moreira et al. 2019; Mowery et al. 2021). Boldness and exploratory behaviour frequently co‐occur in successful invaders and are associated with increased dispersal, enhanced mate locating, and the spread of invasive populations (Baxter‐Gilbert et al. 2021; Chuang and Riechert 2021; Liebl and Martin 2012).

In web‐building spiders, movement ecology plays a central role in reproductive behaviour (Mowery et al. 2022). Adult males leave their webs during the breeding season and must explore their surroundings to locate females (Foelix 2010; Reader 2015). Locomotor behaviours such as running and climbing are key elements of their movement ecology and are often shaped by morphology and habitat structure (Prenter et al. 2010; McGinley et al. 2013). Individual responses to predation risk, such as fleeing, freezing or fighting, may also influence how successfully males search for and find mates (Kralj‐Fišer and Schneider 2012; Johnson and Sih 2007; Smith and Blumstein 2010). These behavioural traits are particularly important in ecologically variable environments such as sand dunes, where movement performance can depend on local habitat conditions (Hann 1990; Maun 2009; Nordberg et al. 2021).

**FIGURE 1:**
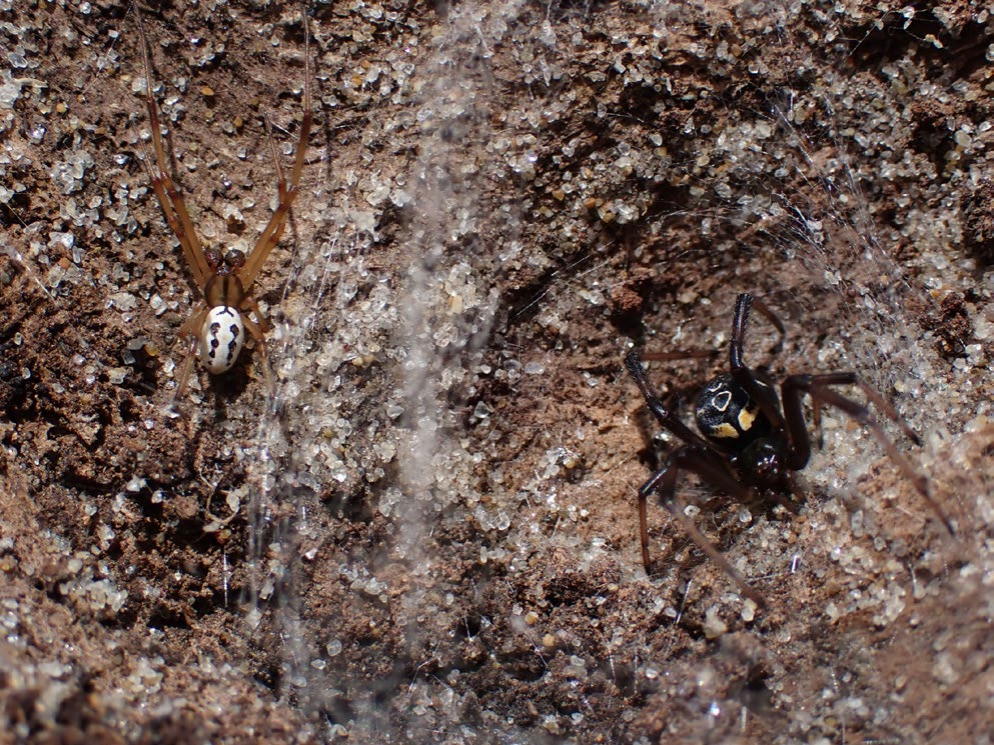
A adult male katipō (left) and subadult female katipō (right) sharing a web under driftwood in the sand dunes.

The katipō ( *Latrodectus katipo* ; Figure 1) (Araneae: Theridiidae) is the only native widow spider in Aotearoa New Zealand. Katipō populations have declined due to widespread sand dune degradation and possible competition with the introduced false katipō ( *Steatoda capensis* ) (Araneae: Theridiidae) (Hann 1990). Although these species may not have direct interactions, such as interbreeding, false katipō may be outcompeting katipō through bolder, more exploratory locomotor behaviours that improve their ability to find mates and recolonise disturbed areas (Hann 1990). Both species are sexually dimorphic, with males exploring to locate females and entering their webs to mate (Forster and Forster 1999). In sand dune habitats, females of both species typically build their webs low beneath driftwood, plant detritus or among native dune vegetation, and usually no more than 100 mm above the sand surface (Griffiths 2001; Hann 1990; Forster and Forster 1999).

Despite concern over katipō decline, few studies have directly compared the movement ecology of adult males from these two species, particularly in northern populations (Forster and Forster 1999; Griffiths 2001; Hann 1990). Given the importance of locomotor behaviours during mate search, investigating variation in movement and exploration may help determine whether traits associated with invasion are contributing to the native species' decline.

In this study, we compared the movement ecology of adult male katipō and false katipō from a sympatric northern dune population. We assessed three behaviours relevant to mate search: exploration, climbing, and running, by using maze, pole and track assays under controlled laboratory conditions. We predicted that false katipō would be more exploratory and faster than katipō, reflecting their broader habitat use and invasive success (Hann 1990). We also expected false katipō, which is on average larger, to run faster than katipō, consistent with the positive correlation between size and speed in other spiders (Brandt and Andrade 2007; Prenter et al. 2010). These comparisons provide insight into how differences in locomotor performance and movement ecology may be facilitating the spread of an introduced species while contributing to the ongoing decline of a threatened native one.

## 2 Materials and Methods

### 2.1 Collection and Husbandry

We collected nine adult male katipō spiders and nine adult male false katipō spiders from sand dunes at Te Ariā Beach, Auckland, Aotearoa New Zealand (36°09′35″ S 174°38′50″ E), during the austral spring and summer (September–February). These seasons were chosen because they are described as the primary reproductive periods for katipō, and the spiders were therefore expected to be easier to find during this time (Forster and Forster 1999). Due to the threatened status of katipō, and our permit restrictions that prohibited returning individuals to the wild, our sample size was restricted to avoid negatively impacting our source population. To maintain comparability between species, the same sample size was used for false katipō. We searched through dune plants such as scrambling pohuehue (*Muehlenbeckia australis*) and kōwhangatara (*Spinifex sericeus*), around naturally occurring logs, and long‐term artificial lizard refuges (length × diameter × height; 500 × 500 × 100 mm onduline sheets) that we placed in the field in December 2022 to assist with collection. Upon collection, we photographed the ventral cephalothorax of each spider next to a scale using an Olympus TG‐5 digital camera. Cephalothorax width was later measured in ImageJ software (Version 1.54a, NIH; Schneider et al. 2012), taken at the point where the second pair of legs (from the front) articulates with the body. Spiders were then held in individual 50 mL plastic vials and transported to Massey University's Albany Campus in an insulated container. In the laboratory, the spiders were maintained in controlled environment rooms at a temperature of 19°C ± 0.5°C, a relative humidity of 60% ± 11% and a reverse 12:12‐h dark cycle (lights off during the day and on at night) with a 1‐h ramp‐up and ramp‐down period to simulate dawn and dusk. Both spider species are nocturnal, and the reverse light cycle in the laboratory allowed the spiders to be tested during the day when the lights were off, simulating night. Individual spiders were housed in 500 mL cylindrical plastic containers (100 × 100 × 150 mm). The container walls were textured by light sanding with 240‐grit sandpaper to allow the spiders to move around the container, as the spiders cannot climb smooth surfaces well. Small holes covered with fine mesh that was glued in place provided ventilation. A hole was made in the side of the container to allow access for feeding and sealed with a tapered rubber stopper measuring 25 mm across the wide end, 20 mm across the narrow end and 45 mm long. Before the first trial day and between trial days, the spiders were fed three wingless fruit flies ( *Drosophila melanogaster* ), and their webs were lightly sprayed with tap water daily. Three wingless fruit flies were provided as a food source before each assay; sufficient food to satiate the spiders.

### 2.2 Test Procedure

Assays were prepared under a red‐light head torch, as both species likely do not see red light (Perampaladas et al. 2008). Three types of exploratory and locomotor behaviours, exploration, running, and climbing, were evaluated across three types of assays: maze, pole and track. These assays simulated key obstacles that spiders face in the sand dunes. The sand dunes where the spiders were collected consist of intertwining dune plants and driftwood, forming a maze‐like environment. Spiders of both species construct their webs in refugia with the superior sheet of the web built 10–100 mm above the ground (Griffiths 2001) and can include kōwhangatara plants, creating pole‐like structures that males can climb. Additionally, bare patches of sand are scattered among vegetated areas. Therefore, spiders must cross these spaces while avoiding potential predators.

Spiders were tested individually, with each spider undergoing three trials per assay, completing one trial of each assay type per assay day. No more than three instances occurred in each assay where individuals were unable to complete an assay in the final replicate (Maze: one katipō and one false katipō; Pole: three false katipō; Track: two false katipō). However, this did not appear to impact the results during analysis. The study spanned 1 week per spider: Day 1—collection, Day 2—rest, Day 3—assays, Day 4—rest, Day 5—assays, Day 6—rest and Day 7—assays. We waited only 1 day after collection for spiders to adjust their circadian rhythm to reduce the number of days they were maintained in the laboratory, which can impact locomotor performance (Trabalon 2022). Spiders appeared to quickly adjust their circadian rhythm, with web‐building and hunting behaviours observed during the second artificial night before the assays. On trial days, the sequence in which individual spiders were tested and the order of the assays each spider was tested in were randomly generated using an online random number generator. The trials were concluded early if the assay goal was observed to be completed, at which point the spider was removed from the assay. If completion could not be determined, the spiders were left until the set assay time had finished. Between each assay, spiders were returned to their housing containers and allowed to rest. The materials were cleaned with 70% ethanol and rinsed with tap water before being wiped down with paper towels and then air dried between assays. All assays were video recorded using either a Canon XA11 or XA20 camcorder under infra‐red light for later analysis.

#### 2.2.1 Maze Assay

**FIGURE 2:**
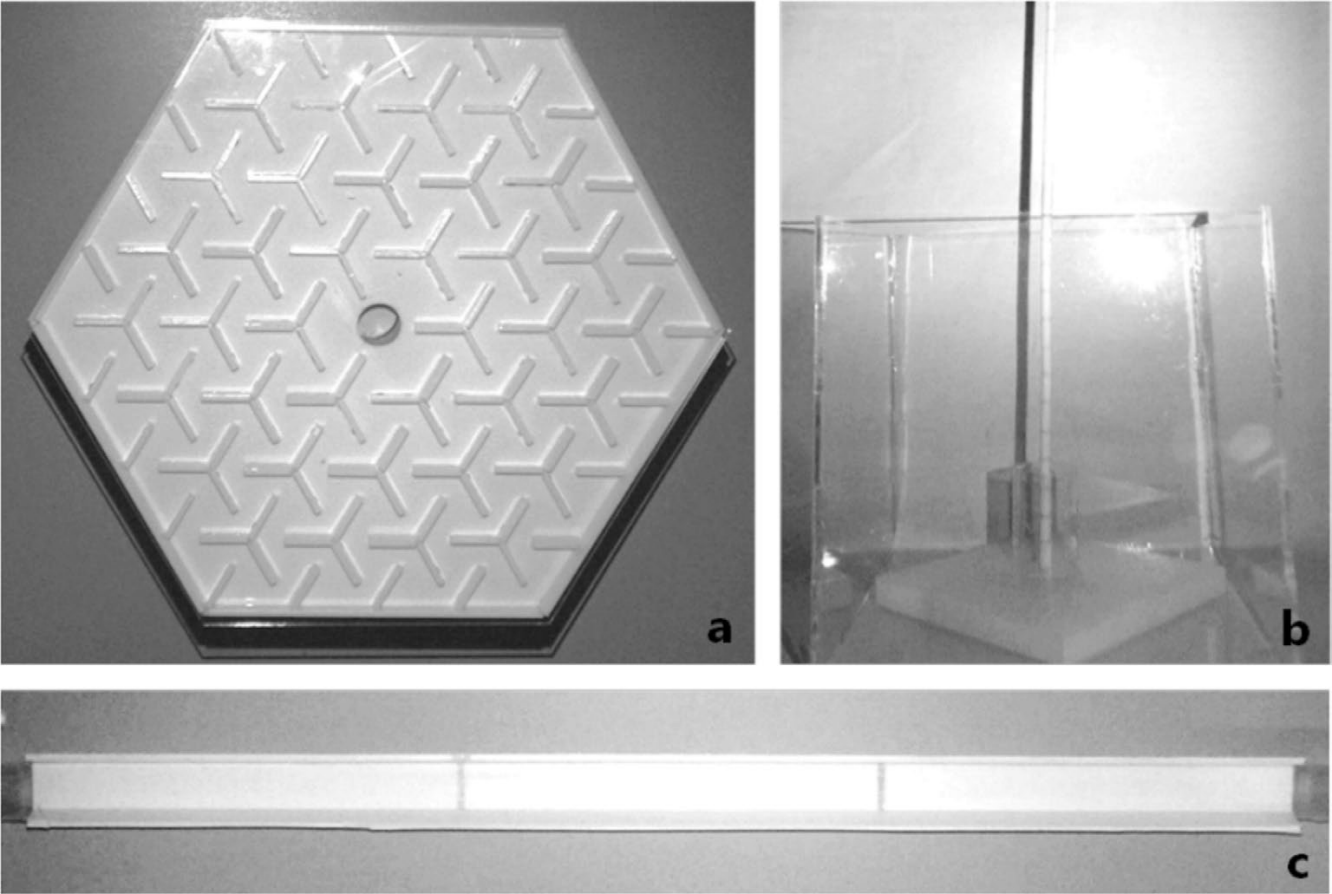
The arenas for the (a) maze assay, the (b) pole assay and the (c) track assay.

We measured how spiders explored a maze (Figure 2a) by observing their entry into and movement throughout the maze. The maze was a hexagonal structure made of white laser‐cut acrylic, 300 mm wide from point to point. At its centre was a 25‐mm diameter hole surrounded by 36 sets of three walls each, arranged in a Y‐shaped pattern. Each wall measured 30 × 3 × 10 mm. The sets were spaced 10 mm apart and uniformly distributed in a radial pattern around the central hole, creating a dense network of pathways for the spiders to navigate. The maze boundaries consisted of walls 150 × 3 × 10 mm extending along each edge of the hexagon. Additionally, 21 independent straight walls extended inward towards the centre from the boundary wall. The entire maze was covered with a clear, hexagonal acrylic sheet. This sheet prevented the spiders from climbing out of the maze and allowed observation and recording of spiders from above. A clear, textured, hollow cylinder, 26 × 26 × 45 mm, was positioned directly below the central hole. The texture was created by sanding the inner surface of the cylinder with 240‐grit sandpaper to provide a rough surface for the spiders to grip. A tapered rubber stopper measuring 25 mm in diameter at the wider end, 20 mm at the narrower end and 45 mm in length was tightly fitted and sealed to the bottom half of the cylinder. For each trial, a spider was placed inside the cylinder on top of the stopper. The spider was given 10 min to exit the cylinder voluntarily into the maze. If the spider did not emerge within this time, we gently encouraged it out by pushing the stopper up until it was flush with the maze base. Once in the maze, the spider was given 15 min to explore. We used the programme AnimalTA (Chiara and Kim 2023) to track each spider's movement and average speed over the 15 min (mm s^−1^).

#### 2.2.2 Pole Assay

We assessed emergence, pole completion, and the time taken to complete the pole climb using a similar experimental design to Brandt and Andrade (2007), but without chasing the spiders with a paintbrush to encourage top speed up the pole (Figure 2b). A cylindrical pole (5 × 5 × 445 mm) was inserted into a 5‐mm diameter hole in the centre of a 3D‐printed rectangular base measuring 100 × 100 × 100 mm. A non‐textured hollow cylinder (26 × 26 × 45 mm) was placed at the base of the pole so that the pole was in the centre of the cylinder. The bottom of the setup was contained within a smooth acrylic box measuring 150 × 150 × 150 mm, ensuring that when the spiders descended from the pole, they would not be able to escape. The trials began by placing the spider into the non‐textured cylinder followed by the insertion of the pole. The spiders were then given 1 h to climb the pole. The assay ended after 1 h, regardless of whether the pole climb was completed by the spider. Emergence was defined as when all tarsi of the spider were touching the pole, and completion of the pole climb was defined by when the front two tarsi touched the top of the pole. If a spider left the pole before completing it, that was defined as emerged but incomplete. The time to complete the pole climb was measured in seconds (s) from when the spider started climbing the pole to when the pole climb was completed.

#### 2.2.3 Track Assay

We assessed the time in seconds to complete the track (Figure 2c) by observing the spiders' running behaviour along a track using similar experimental methods to McGinley et al. (2013). The track measured 500 × 50 × 50 mm and was constructed from white plastic corrugated board, which had been textured for traction by sanding with 240‐grit sandpaper. At the starting point of the track, there was a hollow cylindrical vial placed on its side, measuring 45 × 26 × 26 mm. This vial was also textured and had easily removable lids on both ends. At the end of the track, there was a similar vial, but it had a closed base on the far end and was open at the end connected to the track. Each trial began with a spider placed in the starting vial. The lids of the vial were then removed, and the spider was gently encouraged to run the 500 mm distance to the opposite end using a soft sable hair paintbrush. Spiders were encouraged to run continuously, and if they paused, a gentle touch of the paintbrush to their hind legs was used to prompt them forward. After the first two assays, clear plastic wrap was added to all but one subsequent assay over the track to contain the spiders, as they were found escaping without the plastic wrap and had to be returned to the track approximately where they had exited while moving down. The escape and replacement on the track, as well as the change in chasing method from a short‐handled paintbrush to a long‐handled paintbrush (necessitated by the addition of plastic wrap over the top), which meant that in the first assays the handler's hand was closer to the spider, may have impacted the spiders' time and possibly their behaviour; however, this remains unclear. We recorded the time from when the spider's front tarsi touched the start of the track to when its front tarsi touched the end of the track on the other side.

## 3 Statistics

All statistical analyses were conducted in R version 4.2.1 (R Core Team 2023). Mixed‐effects models were fitted using the lme4 package (Bates et al. 2015), and Cox models were fitted using the survival package (Therneau 2024). We used generalised linear models (GLMs), generalised linear mixed models (GLMMs) and Cox proportional hazards models to analyse our results (Zuur et al. 2009). For each response variable, we constructed a set of candidate models based on biological hypotheses. Size and replicate were retained in all models due to their biological relevance, and interaction terms were tested where interaction was plausible using likelihood ratio tests. We compared models using the corrected Akaike (Akaike 1987) information criterion (AICc) to account for the small sample sizes (Burnham and Anderson 2002). Models within two AICc units of the lowest‐scoring model were considered to have comparable support. In these cases, we selected the simplest model (i.e., with fewer interaction fewer terms) to reduce the risk of overfitting (Arnold 2010). We used the *summary()* function to interpret the direction and strength of individual effects. In addition, we tested interaction terms using likelihood ratio tests (*anova()*) to compare nested models. We also used Anova(type = “II”) from the car package to evaluate the significance of main effects using Wald chi‐square tests. For the Cox model, we assessed the significance of main effects using the *drop1()* function with a chi‐square test, which performs partial likelihood‐based model comparisons suitable for these models. These tests yielded the same conclusions as the AICc‐based model selection, and the *summary()* results, so we report only the AICc results here.

Prior to model fitting, all continuous response variables were assessed using histograms and Q–Q plots and tested for normality using the Shapiro–Wilk test. Where variables were right‐skewed, we evaluated log transformations for better fit. Count data were modelled using either a Poisson or negative binomial distribution depending on the degree of overdispersion, which was assessed using Pearson residuals and the DHARMa package (Hartig et al. 2022). Residual diagnostics for all models were performed using the *simulateResiduals()* function. Model assumptions (e.g., homogeneity, linearity and dispersion) were checked. Multicollinearity among predictors was assessed using the variance inflation factor (VIF), and influential observations were identified using Cook's distance.

Size data were summarised by calculating the minimum, maximum, mean and standard deviation for each species. A GLM with a Gamma distribution and log‐link function was used to compare adult male katipō and false katipō size. Species was included as a fixed effect. The model was fitted using the *glmer()* function.

The simplest models for assay analyses included species, size, and replicate as fixed effects. Individual ID was included as a random effect, unless stated otherwise. Maze emergence, pole emergence and completion were analysed using the *glmer()* function with a binomial distribution. The average speed by an individual in the maze was analysed using the *lmer()* function and a Gaussian distribution. Maze exploration (measured as the total count of virtual squares overlaid on the maze during analysis that were entered by the spider, summed by AnimalTA) was analysed using the *glmmTMB()* function with a negative binomial distribution. Pole emergence analyses also included the interaction between species and replicate as a fixed effect. Track time was analysed using the *glmer()* function with a Gamma distribution and a log‐link function.

Additionally, we determined the proportion of variance explained by the fixed effects in the models (marginal *R* ^2^), and the proportion of variance explained by both the fixed and random effects when relevant (conditional *R* ^2^) using the performance package (Lüdecke et al. 2021). In the maze, pole emergence and pole completion analyses singular fits occurred due to the low variance explained by the random effect (individual ID). Since removing the random effect did not change the fixed effect estimates, we retained individual ID in the model to be consistent with the experimental design, as individuals were tested multiple times.

We compared the time for each species to complete the pole using survival analysis in the survival package (Therneau 2024; Therneau and Grambsch 2000). We used a Cox proportional hazards model with the *coxph()* function, including species, size and replicate as fixed effects, and individual ID as a frailty term. To interpret our results, we followed the recommendations from Muff et al. (2022). *p* values were categorised as follows: 1–0.1 indicated no evidence, 0.1–0.05 indicated weak evidence, 0.05–0.01 indicated moderate evidence and 0.01–0.001 indicated strong evidence of an effect. When presenting estimated means, we include mean ± SD rounded to two decimal places where appropriate. Percentages are presented as whole numbers.

## 4 Results

### 4.1 Size

Adult male katipō were 28% smaller than false katipō (*p* < 0.01; Tables 1 and 2).

**TABLE 1:** Summary of size data (mm) for nine adult male katipō and nine adult male false katipō used in the assays.

Species	Minimum (mm)	Maximum (mm)	Mean ± SD (mm)
Katipō	1035	1302	1138 ± 75
False katipō	1297	1826	1576 ± 175

**TABLE 2:** Generalised linear mixed model (GLMM) assessing if size (mm) varies between species for nine adult male katipō and nine adult male false katipō used in the assays.

Size				
Sample size	9			

Fixed effect	*β*	SE	*t*	*p*
Intercept	0.13	0.02	6.72	**< 0.01**
Species	0.32	0.03	11.58	**< 0.01**
Marginal *R* ^2^	0.72			
AICc	−59.4			

*Note:* Significant *p*‐values < 0.05 are indicated in bold.

### 4.2 Maze Assay

**FIGURE 3:**
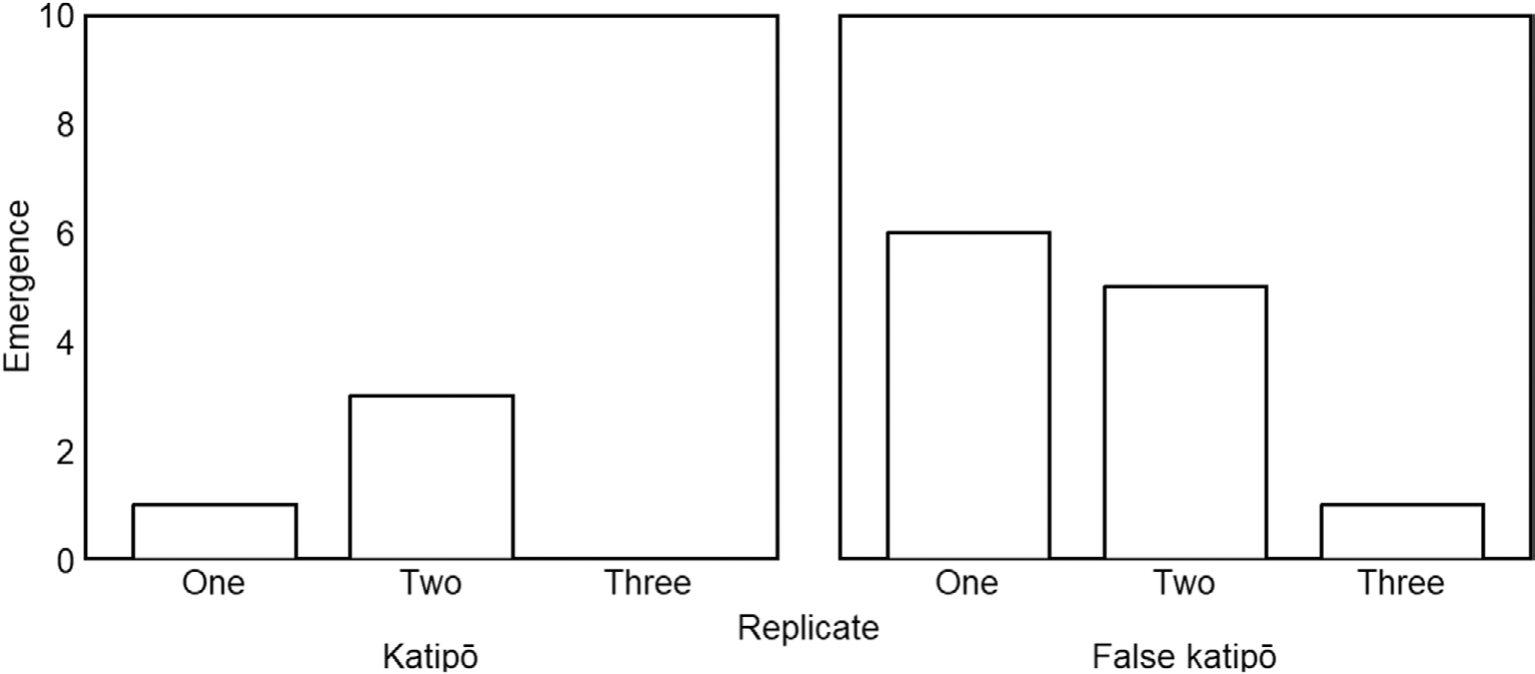
Total number of katipō and false katipō spiders that voluntarily emerged into the maze assay, grouped by replicate. Nine adult males of each species were tested; three replicates were planned per individual and all available replicates were analyzed.

**FIGURE 4:**
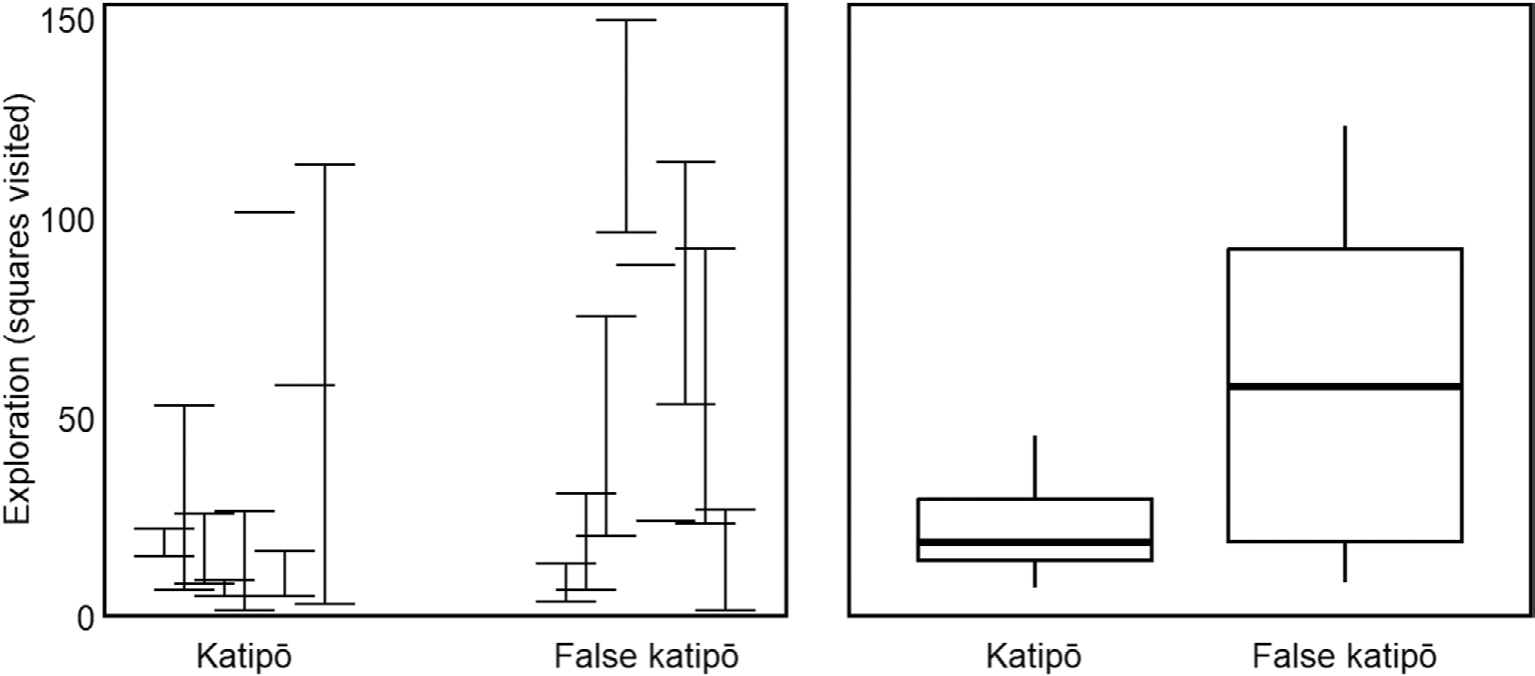
Exploration (count of squares visited) in the maze by adult male katipō and false katipō. Data are from nine individuals per species. Three replicates were planned per individual; all available were analyzed. The left panel shows each individual's mean ± SD. The right panel is a box‑and‑whisker plot: The thick line is the median, the box is the interquartile range (IQR) and the whiskers extend to the most extreme values within 1.5 × IQR.

We found strong evidence that adult male false katipō are more likely to emerge into the maze than adult male katipō (*p* < 0.01, Figure 3 and Table 3). There was a moderate effect of replicate on emergence (*p* < 0.05), with more individuals of both species emerging in the first two replicates compared to the final replicate (Figure 3 and Table 3). We found moderate evidence (*p* < 0.03) that small size was associated with increased emergence (Table 3). Weak evidence supported that false katipō explored more of the maze than katipō (*p* < 0.06, Figure 4 and Table 3). The mean (±SD) exploration in the maze was 27 ± 24 squares for katipō and 61 ± 36 squares for false katipō. There was a no effect of size or replicate on exploration behaviour (Table 3). We found no evidence that katipō and false katipō moved around the maze at different speeds (katipō: 0.14 ± 0.03 mm s^−1^; false katipō: 0.17 ± 0.04 mm s^−1^; Table 3). Size and replicate did not affect average speed for either species (Table 3).

**TABLE 3:** Generalised linear mixed models (GLMMs) assessing the factors influencing emergence (proportion of individuals), average speed (mm s^−1^) and exploration (count of squares entered) in the maze assay.

Maze assay	Emergence				Exploration			Average speed				
Sample size	9				9				9			

Random effects	Variance	SD	*χ* ^2^	*p*	Variance	SD	*χ* ^2^	*p*	Variance	SD	*χ* ^2^	*p*
ID	< 0.01	< 0.01	0.00	1.00	0.40	0.63	5.57	**0.02**	0.00	0.03	23.35	**< 0.01**

Fixed effect	*β*	SE	*z*	*p*	*β*	SE	*z*	*p*	*β*	SE	*t*	*p*
Intercept	6.05	3.13	1.94	**0.05**	4.49	1.50	2.98	**< 0.01**	0.01	0.09	0.11	0.91
Species	4.09	1.37	2.99	**< 0.01**	1.22	0.65	1.88	0.06	0.03	0.04	0.85	0.41
Size	−5.34	2.50	−2.14	**0.03**	−1.02	1.26	−0.81	0.42	0.08	0.07	1.12	0.28
Replicate	−0.98	0.50	−1.99	**0.05**	−0.11	0.14	−0.80	0.42	< 0.01	0.01	0.12	0.90
Marginal *R* ^2^	0.39				0.19				0.23			
Conditional *R* ^2^					0.58				0.38			
AICc	58.90				484.90				−103.50			

*Note:* Significant *p*‐values < 0.05 are indicated in bold. Emergence has no conditional *R* ^2^ because the random effect (ID) has near‐zero variance. Three replicates per individual were planned; all replicates were included in the analysis.

### 4.3 Pole Assay

**FIGURE 5:**
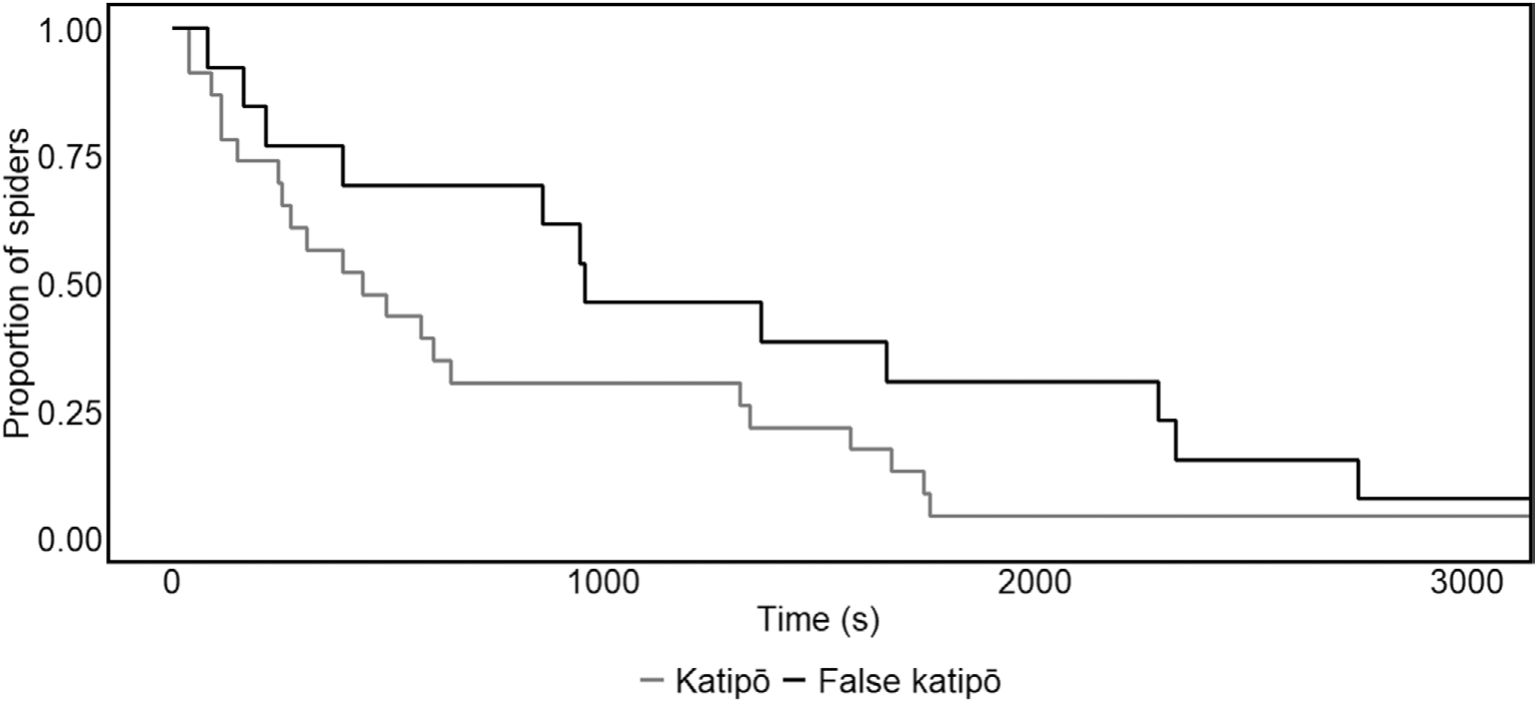
Latency to complete the pole assay for nine adult male katipō and nine adult male false katipō. Three replicates were planned per individual and all available replicates were analyzed. The *y*‑axis shows pole‑completion probability (the proportion yet to finish), and the *x*‑axis shows time (s) to completion; trials were capped at 1 h.

There was no evidence of an effect of species, size, or replicate on whether individuals emerged or completed the pole climb (Table 4). In pooled data across all replicates, 96% of katipō emerged and 85% completed the climb, while 69% of false katipō emerged and 54% completed the climb. Katipō spiders took a mean (±SD) completion time of 833 ± 836 s for the pole climbs, whereas false katipō took 1296 ± 1097 s. There was no evidence that species, size, or replicate influenced pole completion time (Figure 5 and Table 5).

**TABLE 4:** Generalised linear mixed models (GLMMs) assessing the factors influencing emergence and completion in the pole assay. Emergence was when all tarsi of the spider were touching the pole. Completion was when the front two tarci tocuhed the top of the pole.

Pole assay	Emergence				Completion			
Sample size	9				9			

Random effects	Variance	SD	*χ* ^2^	*p*	Variance	SD	*χ* ^2^	*p*
ID	< 0.01	< 0.01	0.00	1.00	< 0.01	< 0.01	0.00	1.00

Fixed effect	*β*	SE	*z*	*p*	*β*	SE	*z*	*p*
Intercept	56.01	868.90	0.06	0.95	1.99	2.66	0.75	0.45
Species	−53.53	868.89	−0.06	0.95	−0.97	1.16	−0.83	0.40
Size	−2.36	2.35	−1.00	0.32	−1.55	2.20	−0.70	0.48
Replicate	−17.08	289.63	−0.06	0.95	0.84	0.46	1.83	0.07
Species × Replicate	17.93	289.63	0.06	0.95				
Marginal *R* ^2^	0.98				0.28			
AICc	45.40				62.80			

*Note:* Three replicates were planned per individual; all replicates were analyzed.

**TABLE 5:** Survival analysis (Cox proportional hazards model) assessing factors influencing the latency to complete the pole assay.

Track assay				
Sample size	9			
Number of events	36			

Fixed effects	Coefficient	SE(coef)	*χ* ^2^	*p*
Species	−0.55	0.63	0.76	0.38
Size	−0.21	1.23	0.03	0.86
Replicate	−0.16	0.22	0.53	0.47
Frailty (Lab ID)				0.94
LRT	*p* = 0.30			
Concordance	0.56 ± 0.06			
AICc	194.74			

*Note:* Three replicates were planned per individual and all replicates were analyzed.

### 4.4 Track Assay

**FIGURE 6:**
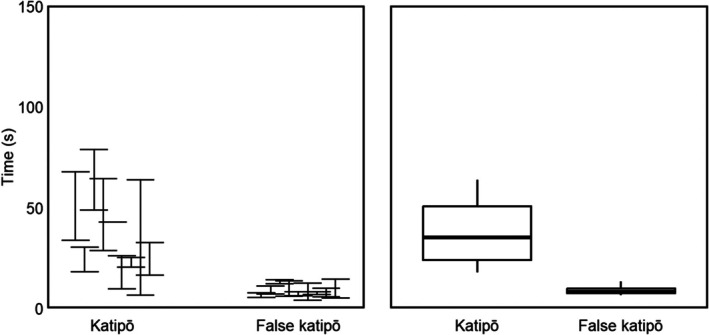
Time to complete the track assay by adult male katipō and false katipō. Replicates were aggregated; three were planned per individual and all available replicates were analyzed. The left panel shows each individual's mean ± SD. The right panel is a box‑and‑whisker plot: The thick line marks the median, the box the interquartile range (IQR) and the whiskers extend to the most extreme values within 1.5 × IQR.

Species had a significant effect on the time to complete the track (*p* < 0.01; Figure 6; Table 6), with false katipō taking a mean (± SD) of 8.8 ± 2.7 s and katipō 41.4 ± 26.6 s. There was no evidence of an effect of replicate or size on the time to complete the track (Table 6).

**TABLE 6:** Generalised linear mixed model (GLMM) assessing factors influencing time to complete the track assay.

Track assay				
Sample size	9			

Random effects	Variance	SD	*χ* ^2^	*p*
ID	0.11	0.33	28.61	**< 0.01**

Fixed effect	*β*	SE	*t*	*p*
Intercept	4.00	0.98	4.06	**< 0.01**
Species	−1.25	0.43	−2.89	**< 0.01**
Size	−0.44	0.85	−0.51	0.61
Replicate	0.03	0.06	0.59	0.55
Marginal *R* ^2^	0.68			
Conditional *R* ^2^	0.82			
AICc	367.40			

*Note:* Significant *p*‐values < 0.05 are indicated in bold. Three replicates were planned per individual and all available replicates were analyzed.

## 5 Discussion

Our findings contribute to movement ecology by showing that false katipō were more exploratory than katipō. False katipō emerged more frequently into the maze compared to katipō, with fewer individuals of both species emerging in the final replicate, potentially due to the spiders' familiarity with the setup. Both species showed similar exploration behaviour in the pole assay. We were unable to compare top running speeds in the track assay because of the different behavioural responses of katipō and false katipō to the paintbrush. There was no difference in average moving speed in the maze between katipō and false katipō, suggesting their average locomotor speed when exploring is similar. Nor was there evidence that morphology (size) within each species affected speed in any assay. Within species, size did negatively correlate with emergence; however, the exact reason remains unclear and would require further experimental investigation.

False katipō explored more areas of the maze compared to katipō. This difference, coupled with increased emergence by the false katipō, leads us to conclude that false katipō have higher levels of exploratory behaviour in the horizontal plane than katipō. Some male katipō needed prompting to emerge, which may have affected their behaviour in the maze. However, the data still suggest a likely behavioural difference between the two species. High levels of exploration could be a key factor in the false katipō's ability to invade new habitats, including empty habitats in sand dunes left open by storm damage, faster than katipō. This has been identified as a potential key threat to katipō (Hann 1990). Species that invade new areas are typically highly exploratory, allowing them to expand their range through increased dispersal rates (Mowery et al. 2021). In contrast, katipō may be confined to a narrower range of habitats due to their less exploratory behaviour (Forster and Forster 1999).

Fewer spiders emerged in the third replicate in the maze assay, which could be attributed to learned behaviour (Punzo 2004). Spiders that have previously explored the maze may have learned that it lacks resources such as shelter, water or potential mates, and therefore saw no value in emerging again. Adult male spiders typically explore areas with a high probability of encountering females and tend to avoid areas where females are absent (Collett 1996; Punzo 2004; Singer and Riechert 1994). If this is a learned behaviour, it suggests that these spiders can assess and remember the utility of an environment, adjusting their exploratory efforts accordingly.

Contrary to our predictions, there was no difference in pole exploration behaviour between the invasive false katipō and endemic katipō. Their similar exploratory behaviour in the pole assay may be attributed to the limited climbing behaviours exhibited in their natural habitats by both species (de Keer et al. 1989; Lapinski and Tschapka 2018; Pekár 2005). As adults, both species construct their webs close to the ground in similar habitats (Forster and Forster 1999; Griffiths 2001). While we did not measure “top speed”, size and speed may still influence the propensity to climb. According to the gravity hypothesis for sexual size dimorphism, smaller size should provide male spiders with an advantage in habitats where successful climbing behaviour is adaptive, as climbing speed decreases with increasing size, and pole climbing speed correlates with size (Moya‐Laraño et al. 2002). However, katipō and false katipō do not appear to be strong climbers in general, and katipō were not observed to climb faster than false katipō despite their smaller size. Furthermore, the lack of a within‐species effect of size on pole climbing completion time is similar to studies on top speed in other spider species, such as the orchard orbweaver ( *Leucauge venusta* ) (Moya‐Laraño et al. 2007; Brandt and Andrade 2007; Prenter et al. 2010). This suggests that factors other than size, such as behavioural adaptations and environment use, play a more significant role in determining climbing behaviour in these spider species.

False katipō were faster to complete the track assays, but there was no difference in average speed between the species in the maze. Notably, behavioural differences in both the track assay and maze could have influenced these results. When followed with a paintbrush, false katipō consistently ran without interruption, whereas katipō often slowed down or stopped. These behaviours reflect two different types of predator avoidance strategies: continuous running and intermittent crouching (Kralj‐Fišer and Schneider 2012; Lohrey et al. 2009; Johnson and Sih 2007). In the maze, false katipō were more exploratory than katipō. Although greater mass can lead to faster individuals (Brandt and Andrade 2007; Prenter et al. 2010), this effect was only seen between species in the track assay and not in the maze, and it was not observed within species. While katipō are possibly slower than false katipō because they are on average 28% smaller and are likely to have a smaller postural locomotor gait, we suggest that these size differences are unlikely to fully explain the differences in running speed observed between the two species in this study. Instead, the running differences are more likely attributed to their respective behavioural adaptations.

While our results should be interpreted with caution due to the relatively small sample size, we suggest that false katipō are more exploratory in the horizontal plane but exhibit similar vertical exploration behaviours to the katipō. From a movement ecology perspective, the higher exploratory behaviour in false katipō suggests an adaptation that enables them to inhabit a broader range of habitats and may facilitate rapid invasion of vacant microhabitats (Hann 1990). Katipō and false katipō differed in the time taken to complete the track assay, which we attribute to behavioural differences rather than size. Though we did not aim to test behavioural differences in response to being chased down the track with a paint brush, the behavioural differences remain intriguing and may be due to differences in anti‐predator behaviour. Further study could provide more insights into these behaviours.

## Author Contributions

**James J. Roberts:** conceptualization (equal), data curation (equal), formal analysis (equal), investigation (equal), methodology (equal), visualization (equal), writing – original draft (equal), writing – review and editing (equal). **Anne Wignall:** conceptualization (equal), formal analysis (equal), methodology (equal), supervision (equal), writing – review and editing (equal). **Dianne H. Brunton:** conceptualization (equal), supervision (equal), writing – review and editing (equal).

## Conflicts of Interest

The authors declare no conflicts of interest.

## Supporting information


**Data S1:** ece372232‐sup‐0001‐Supinfo1.csv.

**Data S2:** ece372232‐sup‐0002‐Supinfo2.pdf.

## Data Availability

The data that support the findings of this study are openly available in Dryad at https://doi.org/10.5061/dryad.wpzgmsc09.
